# Prenatal Immune Challenge Is an Environmental Risk Factor for Brain and Behavior Change Relevant to Schizophrenia: Evidence from MRI in a Mouse Model

**DOI:** 10.1371/journal.pone.0006354

**Published:** 2009-07-24

**Authors:** Qi Li, Charlton Cheung, Ran Wei, Edward S. Hui, Joram Feldon, Urs Meyer, Sookja Chung, Siew E. Chua, Pak C. Sham, Ed X. Wu, Grainne M. McAlonan

**Affiliations:** 1 Department of Psychiatry, University of Hong Kong, Pokfulam, Hong Kong Special Administrative Region (S.A.R.), China; 2 Centre for Reproduction Growth and Development, University of Hong Kong, Pokfulam, Hong Kong Special Administrative Region (S.A.R.), China; 3 Laboratory for Biomedical Imaging and Signal Processing, University of Hong Kong, Pokfulam, Hong Kong Special Administrative Region (S.A.R.), China; 4 Laboratory of Behavioural Neurobiology, Swiss Federal Institute of Technology Zurich (ETH), Schwerzenbach, Switzerland; 5 Department of Anatomy, University of Hong Kong, Pokfulam, Hong Kong Special Administrative Region (S.A.R.), China; 6 State Key Laboratory for Brain and Cognitive Sciences, University of Hong Kong, Pokfulam, Hong Kong Special Administrative Region (S.A.R.), China; University of Queensland, Australia

## Abstract

**Objectives:**

Maternal infection during pregnancy increases risk of severe neuropsychiatric disorders, including schizophrenia and autism, in the offspring. The most consistent brain structural abnormality in patients with schizophrenia is enlarged lateral ventricles. However, it is unknown whether the aetiology of ventriculomegaly in schizophrenia involves prenatal infectious processes. The present experiments tested the hypothesis that there is a causal relationship between prenatal immune challenge and emergence of ventricular abnormalities relevant to schizophrenia in adulthood.

**Method:**

We used an established mouse model of maternal immune activation (MIA) by the viral mimic PolyI:C administered in early (day 9) or late (day 17) gestation. Automated voxel-based morphometry mapped cerebrospinal fluid across the whole brain of adult offspring and the results were validated by manual region-of-interest tracing of the lateral ventricles. Parallel behavioral testing determined the existence of schizophrenia-related sensorimotor gating abnormalities.

**Results:**

PolyI:C-induced immune activation, in early but not late gestation, caused marked enlargement of lateral ventricles in adulthood, without affecting total white and grey matter volumes. This early exposure disrupted sensorimotor gating, in the form of prepulse inhibition. Identical immune challenge in late gestation resulted in significant expansion of 4^th^ ventricle volume but did not disrupt sensorimotor gating.

**Conclusions:**

Our results provide the first experimental evidence that prenatal immune activation is an environmental risk factor for adult ventricular enlargement relevant to schizophrenia. The data indicate immune-associated environmental insults targeting early foetal development may have more extensive neurodevelopmental impact than identical insults in late prenatal life.

## Introduction

The aetiology of complex neurodevelopmental brain disorders such as schizophrenia and autism is unknown. Epidemiological evidence strongly suggests that maternal infection during prenatal life may contribute to an increased risk of schizophrenia or autism in the offspring [1]–[7] and exposure to prenatal infection has been recently linked to specific neuropathological changes in schizophrenia [8]. A causal relationship in this epidemiological link has received considerable support from several experimental models of prenatal infection and/or immune activation. A multitude of behavioural, cognitive and pharmacological abnormalities has been detected in adult mice and rats following prenatal or neonatal exposure to viral pathogens [9]–[15], the viral mimic polyriboinosinic–polyribocytidilic acid (PolyI:C) [16]–[22], the bacterial endotoxin lipopolysaccharide (LPS) [23], [24] and the pro-inflammatory cytokine interleukin (IL)-6 [25], [26]. The most prevalent hypothesis suggests that infection-induced disruption of prenatal or early postnatal brain development predispose the offspring to long-lasting changes in subsequent brain and behavioural development and ultimately leads to adult neuropathology and associated behavioural changes in adolescence or early adulthood [25], [27].

Schizophrenia is associated with significant, progressive brain morphological changes which become evident by early adulthood [28]. Ventricular enlargement is the most consistently replicated finding in patients with schizophrenia [29] and is already present by first clinical presentation of illness and before exposure to anti-psychotic medication [30]. Cannon and colleagues [31] found that ventricular enlargement and associated reductions in white matter and subcortical volumes were unique to the clinical phenotype of schizophrenia. That is, ventricular enlargement in patients is thought to be due to primary causative factors not shared by relatives with some shared genetic risk. Genetic model fitting on twin and sibling data indicates that ventricular enlargement in schizophrenia is driven by individual-specific environmental influences [32] and patients with largest ventricles have also been shown to have poor prognosis [33], [34]. Considering the known impact of prenatal infection on schizophrenia risk, the specificity of ventriculomegaly to the clinical illness and the suggested environmental influences on ventricular enlargement in this disorder, maternal infection during pregnancy may represent a significant environmental risk factor of ventricular enlargement in the offspring. However, direct evidence for this hypothesis is still missing. Examination of prenatal immune activation effects on ventricular abnormalities and associated schizophrenia-related dysfunctions in adult life is clearly warranted.

Therefore, the present study was designed to test the hypothesis that there is a causal relationship between prenatal immune challenge and changes in ventricular size in the adult offspring. We used a well established mouse model of maternal immune activation (MIA) by the viral mimic PolyI:C, which induces a cytokine-associated viral-like acute phase response in mammalian organisms [35]. Prenatal PolyI:C exposure in mice and rats is known to induce a wide spectrum of brain and behavioral dysfunctions implicated in schizophrenia and/or autism (for a review see [36]). Interestingly, recent experimental investigations using the PolyI:C model of MIA have highlighted that the precise timing of MIA is a critical determinant of the specificity of the long-term brain and behavioral pathology emerging in the offspring [36]–[38]. This parallels the human epidemiological findings that the strength of the association between prenatal infection and adult psychotic disorders appears to be critically influenced by the precise timing during prenatal life. In contrast to the data obtained by initial retrospective epidemiological research designs [39]–[42], recent epidemiological studies using serologic markers of maternal infection suggest that infections during the early stages of pregnancy (i.e. in the first trimester of human pregnancy) have a stronger impact on schizophrenia risk in the offspring compared to second or third trimester infections [1], [43]–[45]. However, it is unknown whether a postulated neurodevelopmental impact of early *versus* middle/late prenatal infections on ventricular abnormalities in later life would show a similar temporal dependency.

In order to directly address this issue, we compared the effects of MIA in early (gestation day [GD] 9) and late (GD17) gestation on ventricular abnormalities in the offspring. We adopted an *in-vivo* volumetric magnetic resonance imaging (MRI) approach to help surmount some of the difficulties associated with *ex-vivo* analyses. For example, *ex-vivo* analyses using perfusion-fixation can chemically alter intracellular and extracellular fluid dimensions and subsequent histological processing causes tissue dehydration and mechanical deformation [46]. The *in-vivo* method also has the advantage of allowing further live testing of the animal after scanning [47], [48]. Here, we used 2 complementary MRI analyses methods. First we describe the application of automated voxel-based morphometry (VBM) to map the volume of cerebrospinal fluid (CSF) across the whole brain. VBM relies on intensity based-segmentation of brain into distinct tissue classes (namely, grey matter, white matter and CSF) creating tissue class maps for independent, rapid, and exploratory analysis of every volume-element of brain. Recently, VBM has been applied to the *ex-vivo* study of a mouse model of Huntington's disease [49]. In the present study we propose rapid *in-vivo* scanning for VBM analysis of CSF volume in the MIA mouse model, using segmentation techniques that do not require priori information but instead use manual region-of-interest (ROI) tracing of the lateral ventricles to confirm VBM findings. We predicted that, in addition to the behavioural phenotype, early MIA would be ‘worse’ than later MIA in terms of gross brain anatomical disruption leading to ventricular enlargement.

## Results

### Early but not late prenatal immune challenge leads to enlarged lateral ventricles in adulthood

**Figure 1:**
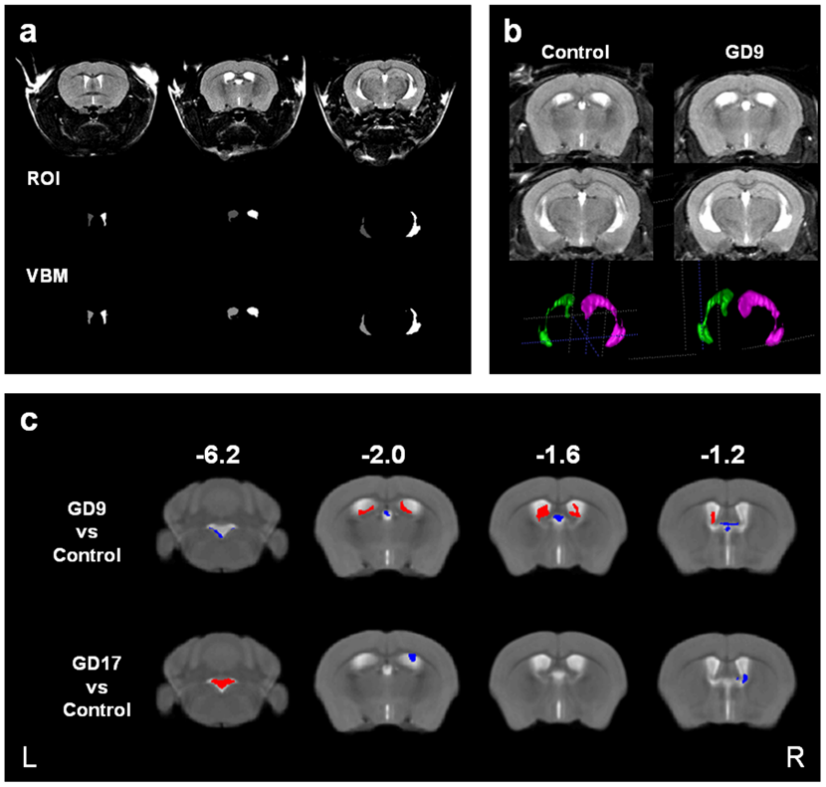
CSF space quantified using VBM and ROI methodology. *a.* Overlap in lateral ventricles quantified using voxel-based morphometry (VBM) and region-of-interest (ROI) methodology showing an animal with the lowest agreement (Dice = 0.92 both sides) between the manual delineation and automatic segmentation of ventricles. Left ventricle in grey; right ventricle in white. *b.* Region-of-Interest defined lateral ventricles. Raw images and the 3D rendered lateral ventricles of a mouse from the control and one from the GD9 group. *c.* Voxel-wise analyses of CSF in adult mice exposed to prenatal challenge at GD9 compared to control and GD17 compared to control. Red indicates a significantly greater likelihood of finding CSF; Blue indicates a significantly lower likelihood of finding CSF.

The volumetric analyses of *in-vivo* MRI data showed that prenatal immune activation in early (GD9) or late (GD17) gestation did not significantly change total brain volume (F_(2,19)_ = .162, p<.851), grey matter (F_(2,19)_ = .017, p<.98) or white matter volume (F_(2,19)_ = .136, p<.87) relative to prenatal control treatment. However, the application of VBM to map the volume of CSF showed greater CSF volumes in the lateral ventricles of offspring exposed to MIA on GD9 compared to control offspring (**Figure 1**). Importantly, the impact of prenatal immune challenge on CSF volumes in the lateral ventricles was clearly restricted to MIA in early gestation because no significant changes in lateral ventricular CSF volumes were apparent in offspring exposed to MIA in late gestation (i.e., on GD17) relative to controls (**Figure 1**). Instead VBM indicated an expansion in 4^th^ ventricle volume in offspring exposed to prenatal challenge in late gestation. ANOVA of region of interest measures supported this effect of prenatal treatment on lateral ventricle volume (F(2,19) = 5.39, p<.01) and subsequent post-hoc analyses confirmed lateral ventriculomegaly in offspring exposed to MIA on GD9 relative to control offspring (see **Tables 1** **,** **2** and **Figure 1**).

**Table 1:** Mean volumes of lateral ventricles and tissue compartments.

Group	Grey	White	G/W	Total Brain Volume	Left Ventricle	Right Ventricle	L/WBV	R/WBV
	(mm^3^)	(mm^3^)		(mm^3^)	(mm^3^)	(mm^3^)		
Control	188.3±15.0	127.0±12.8	1.50	330.4±9.9	2.44±.24	2.43±.28	0.0074	0.0074
GD9	189.7±16.1	124.9±10.5	1.54	328.6±8.4	2.75±.46	2.80±.37*	0.0084	0.0085 †
GD17	188.9±14.7	128.1±12.6	1.49	331.1±6.3	2.38±.38	2.25±.32	0.0072	0.0068

Main tissue compartment volumes and lateral ventricular volumes±Standard Deviation are shown. G/W, ratio of total grey matter to white matter tissue volumes; L/WBV, ratio of left ventricle region-of-interest volume to whole brain volume; R/WBV ratio of right ventricle region-of-interest to whole brain volume.

* Significant difference of Right Ventricle between GD9 and controls (p<.036 two-tailed).

† Significant difference of R/WBV between GD9 and controls (p<.047 two-tailed).

**Table 2:** Voxel-wise comparison of GD9 and GD17 MIA mice with controls.

Location	Cluster Size (mm^3^)	Z score	*p* value	Co-ordinates (mm)		
				X	Y	Z
**GD9>Control**						
Left Lateral Ventricle	0.37	3.11	.001	−0.88	−0.48	−2.64
Right Lateral Ventricle	0.18	2.84	.002	0.96	−0.96	−1.84
**Control>GD9**						
Third Ventricle	0.22	3.73	.000	0.00	−0.32	−3.12
Cerebral Aqueduct	0.13	3.04	.001	−0.08	−4.96	−2.8
Fourth Ventricle	0.26	2.95	.002	−0.24	−5.92	−4.08
Third Ventricle	0.08	2.85	.002	−0.08	−1.44	−6.32
**GD17>Control**						
Fourth Ventricle	0.33	3.39	.000	0.24	−6.48	−3.68
**Control>GD17**						
Right Lateral Ventricle	0.29	3.68	.000	0.8	0.72	−3.6
Left Lateral Ventricle	0.10	3.07	.001	−3.36	−2.16	−4.32
Right Lateral Ventricle	0.17	2.98	.001	1.84	−1.2	−1.68

X, Y, Z co-ordinates are based on the Lambda-Bregma system in the Allen mouse brain atlas. Cluster Size = 0.0768 mm^3^.

### Early but not late prenatal immune challenge disrupts sensorimotor gating in adulthood

**Figure 2:**
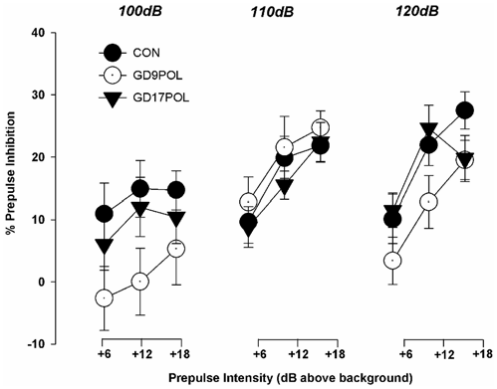
Prepulse inhibition of startle. Behavioral tests in mouse exposed to maternal immune activation at gestation day 9 or 17 (GD9 POL; GD17 POL) compared to controls (CON). Prepulse inhibition of startle (PPI). % PPI =  (pulse-alone - prepulse-plus-pulse)/pulse-alone×100%. A gradual increase in the amount of inhibition was observed as a function of the increasing intensity of the prepulse stimulus. All values are means±SEM.

In this test, a reduction of startle reactivity to the pulse stimulus on prepulse-plus-pulse trials relative to pulse-alone trials constitutes the PPI effect. The analysis of percent PPI (% PPI) confirmed that offspring exposed to MIA on GD9 but not on GD17 displayed reduced sensorimotor gating relative to control offspring (**Figure 2**). A 3×3×3 (group×prepulse intensity×pulse intensity) repeated measures ANOVA showed a significant effect of prepulse intensity [*F*(2, 131) = 11.87, *p*<0.0001] and a significant group difference in %PPI [*F*(2, 131) = 3.56, *p*<0.05]. There was also a significant interaction between pulse intensity and group [*F*(4, 262) = 4.32, *p*<0.01]. Subsequent post-hoc tests verified that the overall group difference in %PPI was driven by a significant impairment in PPI in the GD9 PolyI:C compared to control group (*p*<0.05). This PPI deficit in GD9 PolyI:C animals was evident in the pulse 100 and pulse 120 conditions (*p*<0.05). There was no difference in %PPI between GD17 PolyI:C and control groups (**Figure 2**). There were no significant group differences in startle reactivity, startle habituation or prepulse-elicited reactivity (data not shown). Hence, the PPI attenuating effects of prenatal PolyI:C exposure in early gestation cannot be accounted for by changes in general startle reactivity and/or prepulse detection, reflecting a genuine disruption of sensorimotor gating.

## Discussion

The present study provides the first piece of evidence supporting the hypothesis that prenatal immune activation may be a relevant environmental risk factor for ventricular enlargement in adulthood. Using an experimental model of MIA by the viral mimic PolyI:C, our results demonstrate a casual relationship between maternal exposure to a viral-like acute phase response during early gestation and the subsequent emergence of enlarged lateral ventricles in the adult offspring. Importantly, this relationship appears to be clearly restricted to prenatal immunological insults taking place during early foetal development. This is because no significant changes in lateral ventricular size were apparent in offspring exposed to MIA in late gestation (**Figure 1**). Hence, prenatal immune challenge in early gestation has a greater impact on the development of lateral ventricular abnormalities compared with immune activation at late stages of pregnancy. Our parallel behavioral analysis further confirmed that MIA in early gestation leads to a wider spectrum of schizophrenia-related abnormalities in the adult offspring compared to identical MIA in late gestation: Adult mice prenatally exposed to PolyI:C on GD9 displayed PPI deficits whereas offspring subjected to PolyI:C exposure on GD17 did not. Together, our findings support the hypothesis that early prenatal immune activation in general, and exposure to a viral-like acute phase response in particular, exert a more extensive neurodevelopmental impact in terms of schizophrenia-related brain and behavioral abnormalities compared with immunological insults taking place later in gestation.

The early and late gestational windows selected in the present study (i.e., GD9 and GD17 in the mouse) correspond roughly to the middle of the first and second trimester of human pregnancy respectively [50]. Hence, when extrapolating the present findings to human epidemiological data, our results corroborate recent epidemiological studies using serologic verification of the maternal infection to suggest that infections during the early stages of pregnancy (i.e. in the first trimester of human pregnancy) are associated with the highest risk of schizophrenia and related disorders [1], [3], [43]–[45], [51]. We acknowledge that prenatal immune activation is not the only possible cause of ventricular enlargement, and other rodent models of schizophrenia have shown ventricular enlargement [52]. However, we believe the present experiments do highlight the importance of immune exposure in what is a multifactorial and complex condition. A clear relationship between prenatal infection and risk of ventricular enlargement in schizophrenia has not been established yet in human literature. The present experimental findings may therefore serve to encourage further exploration of possible infectious aetiologies of ventricular abnormalities in schizophrenia and related disorders.

In an elegant study fractionating the contribution of individual cytokines to the behavioural phenotype following MIA, Smith and co-workers [26] found IL6 was a key trigger to neurodevelopmental disruption. In addition to its role in inflammation, IL6 activates the JAK-STAT signal cascade which has an important role in embryonic development, neurogenesis and gliogenesis [53], [54]. Interestingly, the IL6 receptor is highly expressed in the ganglionic eminence (GE) of the germinal cells lining the ventricles. This germinal layer gives rise to neurons and glia during early and mid fetal life and the GE component contains the precursor cells of the striatum and lasts much longer than other proliferative zones of the embryonic brain [55]. It is therefore postulated that activation of the IL6-R of the immature cells of the periventricular zone in early fetal life somehow alters the development and migration of neurons particularly in the striatum, and hence impacts upon the surrounding ventricular space. This is consistent with evidence pointing to disorder of neuronal migration in schizophrenia [56]–[58], disrupted connectivity in schizophrenia [59], [60] and striatal and ventricular morphological differences in schizophrenia [29], [30], [61], [62]. Further evidence that the germinal matrix is especially vulnerable to inflammatory mediators comes from observations of subependymal cysts at the head of the caudate following congenital rubella infection [63]. As mentioned earlier, prenatal rubella infection is strongly associated with schizophrenia [1] and subependymal cysts have also been reported in children with autism who had congenital infection [64].

Our results raise a question about the relevance of late pregnancy MIA to neurodevelopmental disorders. At first glance the absence of ventricular enlargement in GD17 mice appears to shifts the specificity of this exposure away from schizophrenia at least. However, prenatal exposure of mice to human influenza virus on GD18 alters the expression of genes associated with schizophrenia and autism such as Sema3a, Trfr2 and Vldlr [65] and both GD9 and GD18 exposures cause up-regulation of the transcription factor Foxp2 [65], [66] which has been linked with schizophrenia [67] and autism [68]. Therefore one explanation of these findings is that the effect of MIA at GD17 is indeed simply milder than at GD9 [36], that this exposure is sufficient to cause some but not all of the neurodevelopmental phenotype relevant to schizophrenia.

Another possible explanation is that the outcome of MIA on GD17 translates to a different subtype of schizophrenia. Patients with schizophrenia may be categorized on the basis of their memory deficits and associated brain morphology [69]. Those with primarily encoding/storage difficulties or ‘cortical’ pattern deficits are in the minority and have isolated temporal lobe pathology. The majority have a ‘subcortical’ pattern of retrieval difficulties associated with ventricular enlargement and frontal grey matter reductions [69]. Relevant to this discussion are our previous results [38] showing impaired spatial working memory in mice exposed to MIA on GD17, not GD9. Spatial working memory depends upon intact temporal lobe circuits [70], [71], thus the GD17 mice appear to have a degree of commonality with the ‘cortical’ subgroup of patients with schizophrenia who have ‘working’ memory deficits, but no ventricular enlargement. In contrast the GD9 group with enlarged ventricles fits the subcortical or fronto-striatal schizophrenia phenotype described by Turetsky and colleagues [69] or the psychotic cohort with enlarged ventricles sampled in the study by Cannon et al [31]. A PPI impairment restricted to the GD9 ‘ventricular enlargement’ group also fits well with the evidence that sensorimotor gating has a strongly frontal-striatal basis [72]–[74]. Indeed the PPI impairment in schizophrenia has recently been shown to correlate with lower grey matter volumes in dorsolateral prefrontal, middle frontal and the orbital/medial prefrontal cortices [75] and is associated with activation abnormalities in fronto-striatal circuits [72]. Unfortunately it is not known if patients with schizophrenia categorized on the basis of memory impairment can also be distinguished on the basis of PPI impairment, but a prediction that this minority of ‘cortical’ patients have less PPI impairment, would be interesting to test.

Complicating this interpretation is the issue of autism. Autism has been suggested to lie on the same spectrum as schizophrenia with individuals with schizotypy also having a significant number of autistic traits [76]. People with autism spectrum have a strong family history of schizophrenia and bipolar disorder [77] and some children with autism go on to develop schizophrenia in later life. People with Asperger's syndrome have higher scores on measures of paranoia than healthy controls [78] and ‘negative’ symptoms in Asperger's syndrome appear to respond to the antipsychotic risperidone [79]. Together these overlapping observations support the possibility that autism disorders and schizophrenia share causal influences. Although PPI is generally thought to be disrupted in higher functioning individuals with autism spectrum [80], [81], others have found limited evidence for sensorimotor gating abnormalities in a broader sample of ages and abilities [82]. The mice exposed to MIA at GD 17 without PPI deficits therefore appear to better reflect the cohort in this latter study.

The VBM analysis generated an unpredicted effect of late MIA on 4^th^ ventricle volume. This would not have been identified from a ROI analysis alone and highlights the advantage of a whole-brain analytic method in uncovering unexpected, fresh information. What exactly 4^th^ ventricular enlargement following late MIA means will be important to explore. An adjacent loss of regional cerebellar volume might contribute to this result and discrete regions of the cerebellar vermis have been reported to be smaller in schizophrenia despite no overall difference in cerebellar volume [83]. The evidence for cerebellar abnormalities in autism spectrum is extensive and convincing [84]–[88], and changes are likely to be progressive [89], [90]. Potentially the timing of a prenatal event, either genetic or environmental or indeed epigenetic, critically influences phenotypic outcome along a schizophrenia-autism spectrum in later life. Our future work will focus on validating the segmentation of other tissue class compartments segmented in VBM in order to map the full impact of prenatal immune challenge at different time points on brain structure and its relevance to specific neurodevelopmental disorders.

The *in-vivo* scanning design adopted in our study offered a number of other advantages. It meant that behaviour and brain structure could be characterized in the same cohort of animals. However, since the behaviour testing could potentially modulate brain and the procedures involved in MRI, including prolonged anesthetic exposure, might influence behaviour testing, we conducted the PPI study in animals with and without MRI. In addition, we avoided confounding effects of fixation and histology approaches on ventricular morphometry with *in-vivo* MRI. The novel application of VBM methods to map the *in-vivo* CSF space in the mouse was validated using manual ROI measurement of the lateral ventricles and we believe the *in-vivo* technique holds particular promise for the study of longitudinal changes during evolution of the disease process. Conventional anti-psychotic and anti-depressant drug treatment prevents the behavioural impact of early MIA [91] and we are now looking at whether ventricular enlargement can also be halted by drug treatment. The VBM analysis methods adopted here add to the potential for rapid throughput of such studies.

A disadvantage of our study is that resource constraints meant that the numbers included were modest, but supplementing the VBM approach with a manual ROI quantification of ventricular volume lends considerable confidence that the effects seen are robust. Lastly, we included only male animals in our study and we cannot say for certain that the results can be extrapolated to females. There is good evidence that ventricular enlargement in schizophrenia is actually greater in females than males with the condition [92] and, as the MIA behavioural phenotype is observed in both male and female offspring [38], we think it is likely that similar ventricular enlargement would be present in the female offspring. Future studies will address the issue of gender.

In conclusion, we show dramatic differences in brain ventricular morphometry in mice exposed to a brief, non-specific immune challenge during early life in utero. We report that the earlier the exposure the poorer the outcome in terms of both behaviour and brain morphometry. The current experiments do not tell us whether the ventricular changes observed are caused directly or indirectly. We suspect they may be an indirect consequence of regional changes in periventricular structures and our on-going studies will explore this in detail. Thus our work provides strong impetus for further investigation of the role of prenatal immune mechanisms in the pathogenesis of schizophrenia and related disorders because such effects are potentially modifiable. It encourages the application of complementary imaging and behavioural techniques to search for causal mechanisms and new treatments in these poorly understood neurodevelopmental disorders.

## Materials and Methods

### Animals

Female and male C57BL6/N were bred and mated by The University of Hong Kong, Laboratory Animal Unit, (LAU). Timed-pregnant mice were held in a 12∶12 h reversed light-dark cycle (lights on at 19:00), and temperature and humidity-controlled (21±1°C, 55±5%) animal vivarium. Animals were maintained under *ad libitum* food and water diet supplied by the LAU. All experiments were approved by the Committee on the Use of Live Animals in Teaching and Research at The University of Hong Kong and every effort was made to minimize the number of animals used and their suffering.

### Prenatal treatment

This followed the procedures previously reported [16], [17], [37]. PolyI:C (potassium salt) was obtained from Sigma Aldrich and dissolved in saline. A dose of 5 mg/kg in an injection volume of 5 ml/kg, prepared on the day of injection was administered to pregnant dams on GD9 and GD17 via the tail vein under mild physical constraint. Control animals were administered 5 ml/kg saline via tail vein on either GD9 or GD17. The animals were returned to the home cage after the injection. The resulting offspring were weaned and sexed at postnatal day (PND) 21. The pups were weighed and littermates of the same sex were caged separately, three to four per cage. Male offspring only were used in the following experiments.

### MRI

*In-vivo* MRI scanning took place at 12-weeks old in a 7 T scanner with a maximum gradient of 360 mT/m (70/16 PharmaScan, Bruker Biospoin GmbH, Germany). Animals were weighed before scanning and were anesthetized during scanning with isoflurane/air mixture at 3% for induction and 1.5% for maintenance via a nose cone. A quadrature RF coil with 23 mm inner diameter was used. A set of scout images [T_2_ – weighted: Effective TE = 38.71 ms, TR = 4614.566 ms, No of Average = 6, Rare Factor = 8, Acquisition Matrix = 256×256, FOV = 25×25 mm, Slice thickness = 0.25 mm, Scan Time = 11 m4 s] in axial orientation were acquired in each animal. This sequence took less than one hour. Preliminary analysis indicated no difference between animals exposed to saline on GD9 (n = 3) or GD17 (n = 5) therefore these mice were combined in a single control group. The final numbers in MRI study were: controls = 8, PolyI:C = 14 (PolyI:C, GD9 = 8, GD17 = 6).

### Region of interest (ROI) measurement

Manual measurements were done using InsightITK-Snap (http://www.itksnap.org/) by a single rater (WR), blind to subject group membership. Total brain volume was measured from a mask that delineates brain tissue from the skull, generated by the semi-automatic, region growing “3D-snake” method. Each of the masks was manually edited for any miscoverage. Lateral ventricles were delineated according to previously described boundaries and landmarks [93]. Voxels within the region of interest were highlighted slice-by-slice with the “Paint bush” tool. Left and right lateral ventricles were labeled separately.

### Voxel based Morphometry (VBM)

Images were preprocessed with SPM2 (http://www.fil.ion.ucl.ac.uk/~spm/) and FSL (http://www.fmrib.ox.ac.uk/fsl/) running on a Linux workstation. The whole preprocessing routine included skull stripping, custom template creation, tissue classification and normalization.

In the first step, the skull was removed from the raw image by subtracting the brain mask created in the previous section. This ‘skull-stripped’ brain was transformed to a standard space by co-registering to a publicly available mouse template [94] using a 12-paramenter affine transformation [95] in SPM2. The normalized brains of all the mice were averaged to create a custom template.

**Figure 3:**
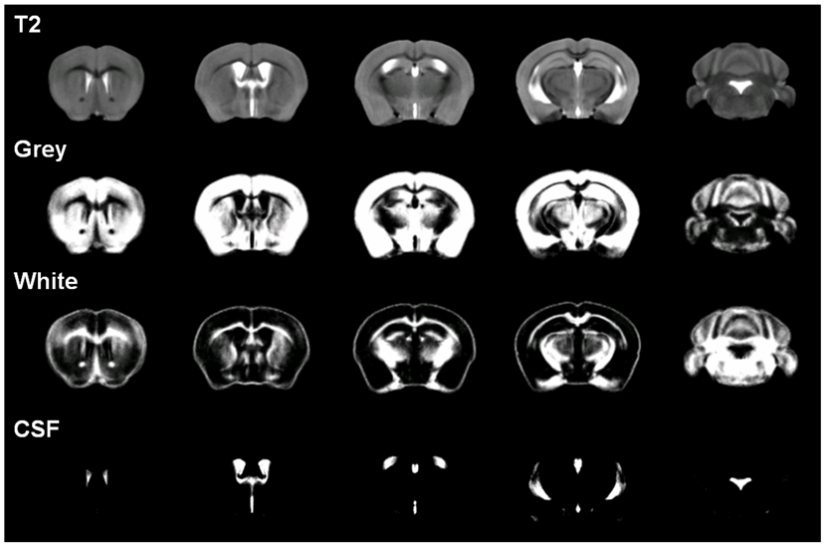
Average T2 image and segmented tissue maps of all the mice used in this study.

The skull-stripped image was segmented into corresponding tissues classes using an intensity based tissue classification kernel [96] in FSL. Since ‘a priori’ information on the probability distribution of different tissue types was not available, the assignment of tissue classes was based solely on voxel intensities. The kernel was set to classify brain tissue into 5 compartments instead of the conventional 3 (grey, white and CSF) for a more refined classification. The extra compartments captured whole brain grey matter comprising cortical, and hippocampal formation grey matter, and whole brain white matter comprising cortical (corpus callosum, internal capsule) and pontine white matter. The tissue maps of the 2 grey matter and 2 white matter compartments were combined to give the more conventional 3-class grey, white and CSF (see **Figure 3**).

In the last step, the skull-free brain was normalized to the custom template using a combination of linear and non-linear transformation [95] in SPM2. The parameters from this transformation were applied to normalize the segmented tissue maps from the previous step. Finally, as ventricular volumes were the focus of the current study, the normalized CSF map was modulated [97] and smoothed with a 0.3 mm FWHM Gaussian kernel for analysis.

Statistical analysis was done in SPM2, using “Single-subject: conditions & covariates” to compare ventriclar volume between different groups. All results were threshold at uncorrected p<.005 (seed) and p<.05 (extension), and a minimum cluster size 150 voxels×(0.08×0.08×0.08) mm/voxel = 0.0768 mm^3^. The co-ordinates of the clusters were reported according to the conventional Lambda-Bregma system in the Allen Mouse Atlas (http://mouse.brain-map.org/atlas/index.html).

In order to validate the segmentation reliability (i.e. to ensure that the VBM result was not driven by misclassifications), regions of left and right lateral ventricles from the automatic procedure were extracted and compared to the manual ROI. The DICE similarity co-efficient [98] was calculated to assess the degree of overlap between manually defined ventricles and ventricles segmented using the VBM routine where: DICE = 2 ( ROI ventricles and VBM ventricles)/(ROI ventricles or VBM ventricles).

The lowest DICE co-efficient between VBM and ROI ventricle measurements was 0.92, and the mean DICE co-efficient between VBM and ROI ventricle measurements was 0.927 for left lateral ventricle and 0.948 for right lateral ventricle indicating substantial overlap between the 2 techniques as shown in **Figure 1**.

### Behavioral phenotyping

Subsequent to the MRI investigations we were interested to confirm the differential impact of early and late MIA on behavioral abnormalities relevant to schizophrenia and related disorders. We assessed the effects of prenatal immune challenge in early (GD9) or late (GD17) gestation, relative to prenatal control treatment, on sensorimotor gating using PPI of the acoustic startle reflex. PPI impairment has been linked to several neuropsychiatric disorders with a presumed neurodevelopmental origin, including schizophrenia [75], [99], [100] and autism [80], [81]. All the animals included in the MRI analysis were tested 1 week after MRI scanning. To rule out any unpredictable effect of MRI on behaviour we extended the sample to include a scan naïve sample prenatally exposed to saline or polyI:C. Behavioral testing was conducted during the dark phase of the light-dark cycle.

#### Prepulse inhibition (PPI) of the acoustic startle reflex

The procedures and testing parameters for evaluation of PPI essentially followed those described previously [101]. In order to exclude the effect of MRI scan on PPI test, we initially compared PPI in a group of mice exposed to saline or polyI:C without MRI scan with groups tested after MRI scan. The results showed no significant effect of scan on PPI in any group (data not shown) therefore mice with and without scan were combined. Final numbers in the PPI experiment were: controls = 18 (8 with MRI scan, 10 without scan), GD9 PolyIC = 13 (8 with scan, 5 without scan) and GD 17 PolyIC = 17 (6 with scan, 11 without scan). Two standard acoustic startle chambers for mice (SR-LAB, San Diego Instruments, San Diego, CA, USA) generated the continuous background noise of 65 dB_A_ and white noise stimuli. Whole body response was transformed to an analogue signal by a piezoelectric unit then digitized and stored. At the onset of the pulse (for pulse alone or prepulse-pulse trials) or prepulse (for prepulse alone trials) 130 readings were taken at 0.5-ms intervals (across 65 ms) and the average amplitude (in arbitrary units) over 65 ms indicated reactivity to the pulse or prepulse stimuli. In a test session lasting approximately 45 min a mixture of pulse-alone, prepulse-plus-pulse, prepulse-alone and no-stimulus (background noise) trials were presented. PPI was calculated as the reduction of startle responses in the prepulse-plus-pulse trials relative to startle in pulse-alone trials. Pulse stimuli were 100, 110, and 120 dB_A_ lasting 40 ms. Prepulse stimuli were 20 ms duration of 6, 12, and 18 dB_A_ units above background respectively with 100 ms stimulus onset asynchrony between prepulse and pulse stimuli on prepulse-plus-pulse trials. Animals were acclimatized for 2 min prior to the first trial. The first 6 trials were 2 pulse only trials of each of the 3 pulse intensities to stabilize the startle response. Then followed 10 blocks of 16 trials in pseudorandom order, each block comprising: 3 pulse-alone trials (100, 110 or 120 dB_A_), 3 prepulse-alone trials (+6, +12, or +18 dB_A_ units above background), 9 combinations of prepulse-plus-pulse trials (3 prepulse options×3 pulse options), and 1 no-stimulus trial (ns), with a variable intertrial interval of a mean of 15 s (10 s to 20 s). The session ended with a final block of 6 pulse-alone trials as in the first block.

### Statistical analysis

Behavioral test data was analyzed using parametric analysis of variance (ANOVA) and analysis of covariance (ANCOVA), followed by post-hoc Scheffe test if appropriate. Where data was not normally distributed, the values were transformed or non-parametric testing adopted. The criterion for statistical significance was set as *p*<0.05. All analyses were conducted using the statistical software SPSS for Windows (version 16). Behavioral and MRI indices from offspring prenatally exposed to control saline treatment at GD9 and GD17 were not different, therefore they were combined to form a single control group. One animal (a GD9 control which failed to thrive postnatally) was excluded from the analyses.

## References

[1] Brown AS, Cohen P, Harkavy-Friedman J, Babulas V, Malaspina D (2001). A.E. Bennett Research Award. Prenatal rubella, premorbid abnormalities, and adult schizophrenia.. Biol Psychiatry.

[2] Brown AS, Schaefer CA, Quesenberry CP, Liu L, Babulas VP (2005). Maternal exposure to toxoplasmosis and risk of schizophrenia in adult offspring.. Am J Psychiatry.

[3] Brown AS (2006). Prenatal infection as a risk factor for schizophrenia.. Schizophr Bull.

[4] O'Callaghan E, Sham P, Takei N, Glover G, Murray RM (1991). Schizophrenia after prenatal exposure to 1957 A2 influenza epidemic.. Lancet.

[5] Sham PC, O'Callaghan E, Takei N, Murray GK, Hare EH (1992). Schizophrenia following pre-natal exposure to influenza epidemics between 1939 and 1960.. Br J Psychiatry.

[6] Chess S (1971). Autism in children with congenital rubella.. J Autism Child Schizophr.

[7] Chess S, Fernandez P, Korn S (1978). Behavioral consequences of congenital rubella.. J Pediatr.

[8] Brown AS, Deicken RF, Vinogradov S, Kremen WS, Poole JH (2009). Prenatal infection and cavum septum pellucidum in adult schizophrenia. Schizophrenia Research 09 January 2009.

[9] Fatemi SH, Earle J, Kanodia R, Kist D, Emamian ES (2002). Prenatal viral infection leads to pyramidal cell atrophy and macrocephaly in adulthood: implications for genesis of autism and schizophrenia.. Cell Mol Neurobiol.

[10] Fatemi SH, Emamian ES, Sidwell RW, Kist DA, Stary JM (2002). Human influenza viral infection in utero alters glial fibrillary acidic protein immunoreactivity in the developing brains of neonatal mice.. Mol Psychiatry.

[11] Shi L, Fatemi SH, Sidwell RW, Patterson PH (2003). Maternal influenza infection causes marked behavioral and pharmacological changes in the offspring.. J Neurosci.

[12] Hornig M, Briese T, Lipkin WI (2001). Bornavirus tropism and targeted pathogenesis: virus-host interactions in a neurodevelopmental model.. Adv Virus Res.

[13] Hornig M, Solbrig M, Horscroft N, Weissenbock H, Lipkin WI (2001). Borna disease virus infection of adult and neonatal rats: models for neuropsychiatric disease.. Curr Top Microbiol Immunol.

[14] Lancaster K, Dietz DM, Moran TH, Pletnikov MV (2007). Abnormal social behaviors in young and adult rats neonatally infected with Borna disease virus.. Behav Brain Res.

[15] Lipkin WI, Hornig M (2003). Microbiology and immunology of autism spectrum disorders.. Novartis Found Symp.

[16] Meyer U, Feldon J, Schedlowski M, Yee BK (2006). Immunological stress at the maternal-foetal interface: a link between neurodevelopment and adult psychopathology.. Brain Behav Immun.

[17] Meyer U, Feldon J, Schedlowski M, Yee BK (2005). Towards an immuno-precipitated neurodevelopmental animal model of schizophrenia.. Neurosci Biobehav Rev.

[18] Meyer U, Murray PJ, Urwyler A, Yee BK, Schedlowski M (2007). Adult behavioral and pharmacological dysfunctions following disruption of the fetal brain balance between pro-inflammatory and IL-10-mediated anti-inflammatory signaling.. Mol Psychiatry.

[19] Patterson PH (2002). Maternal infection: window on neuroimmune interactions in fetal brain development and mental illness.. Current Opinion in Neurobiology.

[20] Watanabe Y, Hashimoto S, Kakita A, Takahashi H, Ko J (2004). Neonatal impact of leukemia inhibitory factor on neurobehavioral development in rats.. Neurosci Res.

[21] Zuckerman L, Rehavi M, Nachman R, Weiner I (2003). Immune activation during pregnancy in rats leads to a postpubertal emergence of disrupted latent inhibition, dopaminergic hyperfunction, and altered limbic morphology in the offspring: a novel neurodevelopmental model of schizophrenia.. Neuropsychopharmacology.

[22] Ozawa K, Hashimoto K, Kishimoto T, Shimizu E, Ishikura H (2006). Immune activation during pregnancy in mice leads to dopaminergic hyperfunction and cognitive impairment in the offspring: a neurodevelopmental animal model of schizophrenia.. Biol Psychiatry.

[23] Romero E, Guaza C, Castellano B, Borrell J (2008). Ontogeny of sensorimotor gating and immune impairment induced by prenatal immune challenge in rats: implications for the etiopathology of schizophrenia.. Mol Psychiatry.

[24] Borrell J, Vela JM, Arevalo-Martin A, Molina-Holgado E, Guaza C (2002). Prenatal immune challenge disrupts sensorimotor gating in adult rats. Implications for the etiopathogenesis of schizophrenia.. Neuropsychopharmacology.

[25] Samuelsson AM, Jennische E, Hansson HA, Holmang A (2006). Prenatal exposure to interleukin-6 results in inflammatory neurodegeneration in hippocampus with NMDA/GABA(A) dysregulation and impaired spatial learning.. Am J Physiol Regul Integr Comp Physiol.

[26] Smith SE, Li J, Garbett K, Mirnics K, Patterson PH (2007). Maternal immune activation alters fetal brain development through interleukin-6.. J Neurosci.

[27] Gilmore JH, Jarskog LF (1997). Exposure to infection and brain development: cytokines in the pathogenesis of schizophrenia.. Schizophr Res.

[28] Ellison-Wright I, Glahn DC, Laird AR, Thelen SM, Bullmore E (2008). The anatomy of first-episode and chronic schizophrenia: an anatomical likelihood estimation meta-analysis.. Am J Psychiatry.

[29] Chua SE, McKenna PJ (1995). Schizophrenia–a brain disease? A critical review of structural and functional cerebral abnormality in the disorder.. Br J Psychiatry.

[30] Chua SE, Cheung C, Cheung V, Tsang JT, Chen EY (2007). Cerebral grey, white matter and csf in never-medicated, first-episode schizophrenia.. Schizophr Res.

[31] Cannon TD, van Erp TG, Huttunen M, Lonnqvist J, Salonen O (1998). Regional gray matter, white matter, and cerebrospinal fluid distributions in schizophrenic patients, their siblings, and controls.. Arch Gen Psychiatry.

[32] Rijsdijk FV, van Haren NE, Picchioni MM, McDonald C, Toulopoulou T (2005). Brain MRI abnormalities in schizophrenia: same genes or same environment?. Psychol Med.

[33] Staal WG, Hulshoff Pol HE, Kahn RS (1999). Outcome of schizophrenia in relation to brain abnormalities.. Schizophr Bull.

[34] Staal WG, Hulshoff Pol HE, Schnack HG, van Haren NE, Seifert N (2001). Structural brain abnormalities in chronic schizophrenia at the extremes of the outcome spectrum.. Am J Psychiatry.

[35] Traynor TR, Majde JA, Bohnet SG, Krueger JM (2004). Intratracheal double-stranded RNA plus interferon-gamma: a model for analysis of the acute phase response to respiratory viral infections.. Life Sci.

[36] Meyer U, Yee BK, Feldon J (2007). The neurodevelopmental impact of prenatal infections at different times of pregnancy: the earlier the worse?. Neuroscientist.

[37] Meyer U, Nyffeler M, Engler A, Urwyler A, Schedlowski M (2006). The time of prenatal immune challenge determines the specificity of inflammation-mediated brain and behavioral pathology.. J Neurosci.

[38] Meyer U, Nyffeler M, Yee BK, Knuesel I, Feldon J (2008). Adult brain and behavioral pathological markers of prenatal immune challenge during early/middle and late fetal development in mice.. Brain, Behavior, and Immunity.

[39] Mednick SA, Machon RA, Huttunen MO, Bonett D (1988). Adult schizophrenia following prenatal exposure to an influenza epidemic.. Arch Gen Psychiatry.

[40] Suvisaari J, Haukka J, Tanskanen A, Hovi T, Lonnqvist J (1999). Association between prenatal exposure to poliovirus infection and adult schizophrenia.. Am J Psychiatry.

[41] Limosin F, Rouillon F, Payan C, Cohen JM, Strub N (2003). Prenatal exposure to influenza as a risk factor for adult schizophrenia.. Acta Psychiatr Scand.

[42] Beydoun H, Saftlas AF (2008). Physical and mental health outcomes of prenatal maternal stress in human and animal studies: a review of recent evidence.. Paediatr Perinat Epidemiol.

[43] Brown AS, Begg MD, Gravenstein S, Schaefer CA, Wyatt RJ (2004). Serologic evidence of prenatal influenza in the etiology of schizophrenia.. Arch Gen Psychiatry.

[44] Babulas V, Factor-Litvak P, Goetz R, Schaefer CA, Brown AS (2006). Prenatal exposure to maternal genital and reproductive infections and adult schizophrenia.. Am J Psychiatry.

[45] Sorensen HJ, Mortensen EL, Reinisch JM, Mednick SA (2008). Association Between Prenatal Exposure to Bacterial Infection and Risk of Schizophrenia.. Schizophr Bull.

[46] Hayat MA (2000). Principles and Techniques of Electron Microscopy: Biological Applications.:.

[47] Wolf OT, Dyakin V, Patel A, Vadasz C, de Leon MJ (2002). Volumetric structural magnetic resonance imaging (MRI) of the rat hippocampus following kainic acid (KA) treatment.. Brain Res.

[48] Wolf OT, Dyakin V, Vadasz C, de Leon MJ, McEwen BS (2002). Volumetric measurement of the hippocampus, the anterior cingulate cortex, and the retrosplenial granular cortex of the rat using structural MRI.. Brain Res Brain Res Protoc.

[49] Sawiak SJ, Wood NI, Williams GB, Morton AJ, Carpenter TA (2009). Voxel-based morphometry in the R6/2 transgenic mouse reveals differences between genotypes not seen with manual 2D morphometry.. Neurobiol Dis.

[50] Clancy B, Kersh B, Hyde J, Darlington RB, Anand KJ (2007). Web-based method for translating neurodevelopment from laboratory species to humans.. Neuroinformatics.

[51] Rehn AE, Rees SM (2005). Investigating the neurodevelopmental hypothesis of schizophrenia.. Clin Exp Pharmacol Physiol.

[52] Torres G, Meeder BA, Hallas BH, Spernyak JA, Mazurchuk R (2005). Ventricular size mapping in a transgenic model of schizophrenia.. Brain Res Dev Brain Res.

[53] Heinrich PC, Behrmann I, Haan S, Hermanns HM, Muller-Newen G (2003). Principles of interleukin (IL)-6-type cytokine signalling and its regulation.. Biochem J.

[54] Zhao B, Schwartz JP (1998). Involvement of cytokines in normal CNS development and neurological diseases: recent progress and perspectives.. J Neurosci Res.

[55] Kostovic I, Judas M (1998). Transient patterns of organization of the human fetal brain.. Croat Med J.

[56] Bunney BG, Potkin SG, Bunney WE (1995). New morphological and neuropathological findings in schizophrenia: a neurodevelopmental perspective.. Clin Neurosci.

[57] Chua SE, Murray RM (1996). The neurodevelopmental theory of schizophrenia: evidence concerning structure and neuropsychology.. Ann Med.

[58] Beckmann H (1999). Developmental malformations in cerebral structures of schizophrenic patients.. Eur Arch Psychiatry Clin Neurosci.

[59] Cheung V, Cheung C, McAlonan GM, Deng Y, Wong JG (2008). A diffusion tensor imaging study of structural dysconnectivity in never-medicated, first-episode schizophrenia.. Psychol Med.

[60] Hoptman MJ, Ardekani BA, Butler PD, Nierenberg J, Javitt DC (2004). DTI and impulsivity in schizophrenia: a first voxelwise correlational analysis.. Neuroreport.

[61] Chua SE, Lam IW, Tai KS, Cheung C, Tang WN (2003). Brain morphological abnormality in schizophrenia is independent of country of origin.. Acta Psychiatr Scand.

[62] Chua SE, Lam IW, Chen EY, Lee PW, Lieh-Mak F (2002). Asymmetric lateral ventricular enlargement in Chinese with 1st episode schizophrenia.. Schizophr Res.

[63] Beltinger C, Saule H (1988). Sonography of subependymal cysts in congenital rubella syndrome.. Eur J Pediatr.

[64] Yamashita Y, Fujimoto C, Nakajima E, Isagai T, Matsuishi T (2003). Possible association between congenital cytomegalovirus infection and autistic disorder.. J Autism Dev Disord.

[65] Fatemi SH, Reutiman TJ, Folsom TD, Huang H, Oishi K (2008). Maternal infection leads to abnormal gene regulation and brain atrophy in mouse offspring: Implications for genesis of neurodevelopmental disorders.. Schizophr Res.

[66] Fatemi SH, Reutiman TJ, Folsom TD, Sidwell RW (2008). The role of cerebellar genes in pathology of autism and schizophrenia.. Cerebellum.

[67] Sanjuan J, Tolosa A, Gonzalez JC, Aguilar EJ, Perez-Tur J (2006). Association between FOXP2 polymorphisms and schizophrenia with auditory hallucinations.. Psychiatr Genet.

[68] Gong X, Jia M, Ruan Y, Shuang M, Liu J (2004). Association between the FOXP2 gene and autistic disorder in Chinese population.. Am J Med Genet B Neuropsychiatr Genet.

[69] Turetsky BI, Moberg PJ, Mozley LH, Moelter ST, Agrin RN (2002). Memory-delineated subtypes of schizophrenia: relationship to clinical, neuroanatomical, and neurophysiological measures.. Neuropsychology.

[70] Kesner RP, Giles R (1998). Neural circuit analysis of spatial working memory: role of pre- and parasubiculum, medial and lateral entorhinal cortex.. Hippocampus.

[71] Galani R, Obis S, Coutureau E, Jarrard L, Cassel JC (2002). A comparison of the effects of fimbria-fornix, hippocampal, or entorhinal cortex lesions on spatial reference and working memory in rats: short versus long postsurgical recovery period.. Neurobiol Learn Mem.

[72] Hazlett EA, Buchsbaum MS, Zhang J, Newmark RE, Glanton CF (2008). Frontal-striatal-thalamic mediodorsal nucleus dysfunction in schizophrenia-spectrum patients during sensorimotor gating.. Neuroimage.

[73] Swerdlow NR, Lipska BK, Weinberger DR, Braff DL, Jaskiw GE (1995). Increased sensitivity to the sensorimotor gating-disruptive effects of apomorphine after lesions of medial prefrontal cortex or ventral hippocampus in adult rats.. Psychopharmacology (Berl).

[74] Japha K, Koch M (1999). Picrotoxin in the medial prefrontal cortex impairs sensorimotor gating in rats: reversal by haloperidol.. Psychopharmacology (Berl).

[75] Kumari V, Fannon D, Geyer MA, Premkumar P, Antonova E (2008). Cortical grey matter volume and sensorimotor gating in schizophrenia.. Cortex.

[76] Esterberg ML, Trotman HD, Brasfield JL, Compton MT, Walker EF (2008). Childhood and current autistic features in adolescents with schizotypal personality disorder.. Schizophr Res.

[77] Ghaziuddin M (2005). A family history study of Asperger syndrome.. J Autism Dev Disord.

[78] Craig JS, Hatton C, Craig FB, Bentall RP (2004). Persecutory beliefs, attributions and theory of mind: comparison of patients with paranoid delusions, Asperger's syndrome and healthy controls.. Schizophr Res.

[79] Rausch JL, Sirota EL, Londino DL, Johnson ME, Carr BM (2005). Open-label risperidone for Asperger's disorder: negative symptom spectrum response.. J Clin Psychiatry.

[80] McAlonan GM, Daly E, Kumari V, Critchley HD, van Amelsvoort T (2002). Brain anatomy and sensorimotor gating in Asperger's syndrome.. Brain.

[81] Perry W, Minassian A, Lopez B, Maron L, Lincoln A (2007). Sensorimotor gating deficits in adults with autism.. Biol Psychiatry.

[82] Ornitz EM, Lane SJ, Sugiyama T, de Traversay J (1993). Startle modulation studies in autism.. J Autism Dev Disord.

[83] Nopoulos PC, Ceilley JW, Gailis EA, Andreasen NC (1999). An MRI study of cerebellar vermis morphology in patients with schizophrenia: evidence in support of the cognitive dysmetria concept.. Biol Psychiatry.

[84] Pierce K, Courchesne E (2001). Evidence for a cerebellar role in reduced exploration and stereotyped behavior in autism.. Biol Psychiatry.

[85] McAlonan GM, Cheung V, Cheung C, Suckling J, Lam GY (2005). Mapping the brain in autism. A voxel-based MRI study of volumetric differences and intercorrelations in autism.. Brain.

[86] Bailey AJ (1993). The biology of autism.. Psychol Med.

[87] Bauman ML, Kemper TL (2003). The neuropathology of the autism spectrum disorders: what have we learned?. Novartis Found Symp.

[88] Carper RA, Courchesne E (2000). Inverse correlation between frontal lobe and cerebellum sizes in children with autism.. Brain.

[89] Bauman ML, Kemper TL (2005). Neuroanatomic observations of the brain in autism: a review and future directions.. Int J Dev Neurosci.

[90] Shi L, Smith SE, Malkova N, Tse D, Su Y (2009). Activation of the maternal immune system alters cerebellar development in the offspring.. Brain Behav Immun.

[91] Meyer U, SpoerrI E, Yee B, Schwarz MJ, Feldon J (2008). Evaluating Early Preventive Antipsychotic and Antidepressant Drug Treatment in an Infection-Based Neurodevelopmental Mouse Model of Schizophrenia.. Schizophrenia Bulletin.

[92] Chance SA, Esiri MM, Crow TJ (2003). Ventricular enlargement in schizophrenia: a primary change in the temporal lobe?. Schizophr Res.

[93] Dorr AE, Lerch JP, Spring S, Kabani N, Henkelman RM (2008). High resolution three-dimensional brain atlas using an average magnetic resonance image of 40 adult C57Bl/6J mice.. Neuroimage.

[94] Chan E, Kovacevic N, Ho SK, Henkelman RM, Henderson JT (2007). Development of a high resolution three-dimensional surgical atlas of the murine head for strains 129S1/SvImJ and C57Bl/6J using magnetic resonance imaging and micro-computed tomography.. Neuroscience.

[95] Ashburner J, Friston KJ (1999). Nonlinear spatial normalization using basis functions.. Hum Brain Mapp.

[96] Smith SM, Jenkinson M, Woolrich MW, Beckmann CF, Behrens TE (2004). Advances in functional and structural MR image analysis and implementation as FSL.. Neuroimage.

[97] Good CD, Johnsrude IS, Ashburner J, Henson RN, Friston KJ (2001). A voxel-based morphometric study of ageing in 465 normal adult human brains.. Neuroimage.

[98] Dice LR (1945). Measures of the amount of ecologic association between species.. Ecology.

[99] Braff DL, Geyer MA, Swerdlow NR (2001). Human studies of prepulse inhibition of startle: normal subjects, patient groups, and pharmacological studies.. Psychopharmacology (Berl).

[100] Swerdlow NR, Light GA, Cadenhead KS, Sprock J, Hsieh MH (2006). Startle gating deficits in a large cohort of patients with schizophrenia: relationship to medications, symptoms, neurocognition, and level of function.. Arch Gen Psychiatry.

[101] Pietropaolo S, Singer P, Feldon J, Yee BK (2008). The postweaning social isolation in C57BL/6 mice: preferential vulnerability in the male sex.. Psychopharmacology (Berl).

